# Understanding delays in the introduction of complementary foods in rural Ethiopia

**DOI:** 10.1111/mcn.13247

**Published:** 2021-09-15

**Authors:** Kalle Hirvonen, Abdulazize Wolle, Arnaud Laillou, Vincenzo Vinci, Stanley Chitekwe, Kaleab Baye

**Affiliations:** ^1^ Development Strategy and Governance Division International Food Policy Research Institute (IFPRI) Addis Ababa Ethiopia; ^2^ Economics Department State University of New York at Albany Albany New York USA; ^3^ Nutrition Section UNICEF Addis Ababa Ethiopia; ^4^ Social Policy Section UNICEF Addis Ababa Ethiopia; ^5^ Center for Food Science and Nutrition. College of Natural Sciences Addis Ababa University Addis Ababa Ethiopia; ^6^ Research Center for Inclusive Development in Africa (RIDA) Addis Ababa Ethiopia

**Keywords:** breastfeeding, child feeding, complementary foods, linear growth, stunting

## Abstract

Age‐appropriate breastfeeding and introduction to complementary foods can shape child feeding practices, ensure adequate energy and nutrient intake and prevent linear growth faltering. This study aimed to assess mothers' and health workers' knowledge of timely introduction to complementary foods and evaluate the relationship between delays in complementary feeding and subsequent linear growth. We conducted two rounds of surveys (March/August 2017) among 249 health workers (*n* = 249) and caregivers (*n* = 2635) of children 6–23 months of age. We collected information about socio‐demographic characteristics, knowledge and practice related to timely introduction to complementary foods. The study was conducted in households from the Productive Safety Net Programme (PSNP) districts, in four highland regions of Ethiopia. Delays in the introduction to complementary feeding were widespread with 53% of children 6–8 months of age not consuming solid, semisolid or soft foods in the past 24 h. After controlling for child, caregiver and household characteristics, children not introduced to complementary foods by 6–8 months had a 0.48 SD lower length‐for‐age *z*‐score at 12–15 months. Caregivers' knowledge was strongly and inversely correlated with untimely introduction of complementary foods in logistic regressions (OR = 0.55, *p* < 0.01). In turn, local health extension worker's knowledge was strongly correlated with caregiver's knowledge. Consequently, frequent and timely visits by health extension workers emphasising not only on what to feed but also when and how to feed a child are needed. Innovative ways of increasing reach, intensity and frequency of nutrition messaging by using the PSNP interactions as an additional point of contact would need to be explored further.

**Key messages**

- Age‐appropriate breastfeeding was common, but delays in the introduction of complementary feeding were widespread
- Delays in complementary feeding are strongly associated with linear growth faltering
- Health workers' knowledge predicts child feeding related knowledge of caregivers
- Knowledge gap of caregivers is strongly correlated with delays in complementary feeding, irrespective of wealth status, education and remoteness

## 1 INTRODUCTION

Child malnutrition, although declining, is still a significant public health concern in many low‐ and middle‐income countries (LMICs) (Victora et al., 2021). In 2019, 144 million children less than 5 years of age were stunted globally (UNICEF, WHO,, & World Bank Group, 2020). The timing of growth faltering coincides with the complementary feeding period (6–23 months), suggesting that accelerating progress requires a much more significant improvement in complementary feeding practices (Victora, de Onis, Hallal, Blossner, & Shrimpton, 2010). Currently, less than a third of children in LMICs meet the recommended complementary feeding practices (Baye & Kennedy, 2020). Therefore, effective interventions that improve complementary feeding as part of the first 1000 days of life, spanning from conception to the second birth day, are needed to prevent stunting and promote healthy child growth (Baye & Faber, 2015; Martorell, 2017).

Similar to global trends (Victora, de Onis, Hallal, Blossner, & Shrimpton, 2010), linear growth faltering in Ethiopia begins to accelerate at six months of age (Golan, Headey, Hirvonen, & Hoddinott, 2019). A recent study has also shown that the highest number of child stunting can be averted by improving complementary feeding (Baye, 2019). With only 12.5% of children 6–23 months of age meeting the minimum dietary diversity (MDD), complementary feeding practices remain suboptimal (CSA & ICF, 2016). The improvements in MDD and the reductions in stunting witnessed over the years were primarily driven by improvements seen in the wealthiest quintile (Baye, 2021). For example, there is a 19—percentage point difference in the stunting prevalence between the poorest (44.6%) and the richest (25.6%) wealth quintile, suggesting that due attention is needed to support the poorest wealth quintile to prevent further widening of inequalities (Baye, Laillou, & Chitweke, 2020). These inequalities in nutritional outcomes could potentially be addressed by integrating locally adapted infant and young child feeding (IYCF) messages to existing social protection programmes (i.e., Productive Safety Net Programme—PSNP) or other platforms operating at scale.

For effective implementation of locally adapted nutrition messaging, the nutrition knowledge of the health workers and their counselling skills are critical (Abebe, Haki, & Baye, 2016). This is especially the case in remote and poor households that primarily rely on the Health Extension Programme (HEP) for nutrition education (Abate, Dereje, Hirvonen, & Minten, 2020). Timing of the nutrition message delivery is also important as it can influence the timing of the adoption of recommended practices. This is critical as both too early or too late introduction to complementary feeding have been associated with adverse outcomes. For example, earlier introduction to complementary foods (<4 months) has been linked to child overweight (Baidal et al., 2016), whereas late introduction has been associated with nutrient deficiencies like those of iron and zinc (PAHO/WHO, 2003). In Ethiopia, delayed introduction of complementary feeding is widespread as only 59% of children 6–8 months of age were reported to have been introduced to complementary feeding in the 2016 Demographic and Health Survey (CSA & ICF, 2016). Whether this delay is linked to food insecurity, nutrition illiteracy or other contextual factors remains unknown. Besides, there is limited understanding of the effectiveness of nutrition education in resource‐constrained settings like in the PSNP districts. Consequently, systematic evaluation of how health workers IYCF knowledge influences caregivers' knowledge and how this in turn translates to IYCF practices and child growth outcomes is needed.

Focusing on understanding delays in the introduction of complementary foods, the present study aimed to assess the knowledge of mothers and health workers and evaluate the association between knowledge, timely introduction of complementary feeding and subsequent linear growth among children residing in poor households located in PSNP districts. Such information can inform the design of effective nutrition education programmes through better integration with existing social protection systems.

## 2 METHODS

### 2.1 Context

This study focused on the four highland regions of Ethiopia: Amhara, Oromia, Southern Nations, Nationalities and Peoples' Region (SNNP‐including the newly formed Sidama region) and Tigray. These regions are predominantly rural and home to more than 85% of the country's population (CSA, 2013). Within these regions, the study focused on chronically food insecure woredas (districts) in which Ethiopia's flagship safety net programme—the Productive Safety Net Programme (PSNP)—operates. With 8 million people benefitting from the programme, PSNP is one of the largest safety net programmes in sub‐Saharan Africa (World Bank, 2018). Beneficiary households receive either cash or food payments against labour intensive public works, while labour constrained households receive unconditional payments. While household food security has been improving in these localities, partly attributable to the PSNP (Berhane, Hirvonen, & Hoddinott, 2016), this has not translated to better nutritional outcomes for infants and young children (Berhane et al., 2020; Berhane, Hoddinott, Kumar, & Margolies, 2015). Chronic undernutrition prevalence among young children in PSNP localities remains high, and intra‐annual fluctuations in child weights relative to their heights are large (Baye & Hirvonen, 2020b). Moreover, child diets in the PSNP districts are extremely monotonous with less than 5% of the children meeting MDD, as defined by the WHO (2008) (Berhane et al., 2020). Moreover, the differences in children's anthropometric measures as well as dietary outcomes between households benefitting from the PSNP and other poor households have been shown to be negligible (Berhane et al., 2020).

The fourth phase of the PSNP introduced a nutrition sensitive design (GFDRE, 2014), aiming to facilitate linkages with health and nutrition services (Bossuyt, 2017). Pregnant or lactating women are temporarily moved from the public works to receive unconditional payments (i.e., direct support) with the idea of reducing women's workload and permitting more time for childcare. Moreover, women are encouraged to attend antenatal care and after delivery regularly visit the local health post with their newborn child. Women participating in public works are expected to attend in nutrition behavioural change communication sessions.

This nutrition sensitive PSNP design is closely aligned with the HEP, a community‐based health service delivery structure led and designed by the government of Ethiopia (Workie & Ramana, 2013). At the community level, the implementation of the HEP relies on health extension workers (HEWs) that take the responsibility in providing basic health care services and promoting health and nutrition‐related messages in rural communities (Abate, Dereje, Hirvonen, & Minten, 2020). By design, each kebele (subdistrict) should have one health post, typically staffed by two female HEWs responsible for reaching approximately 5000 individuals (Lemma & Matji, 2013). HEWs also play a central role in the implementation of the nutrition sensitive activities of the PSNP, particularly with respect to the provision of nutrition‐related behaviour change communication (Bossuyt, 2017).

### 2.2 Data

The data for this secondary analysis came from a large and geographically widespread longitudinal survey of children and their mothers conducted in the four highland regions. The original purpose of the survey was to gather baseline information for an evaluation of nutrition sensitive components of the fourth phase of the PSNP (Berhane et al., 2020). In March 2017, a stratified sample of 88 woredas was randomly drawn from areas in which the PSNP operates in the four highland regions. From each woreda, three enumeration areas (EAs), one from each kebele (subdistrict), were randomly selected. From each of the 264 EA, 10 households with a child less than 24 months were randomly selected into the sample, with approximately 50–50 split between PSNP beneficiary households and poor nonbeneficiary households. In March 2017, 2635 household were interviewed. Out of these 2635 households, 2569 were successfully revisited in August 2017, yielding an attrition rate of less than 3%. Supporting information S1 describes the sampling strategy in more detail.

The survey collected detailed information about household assets, education levels and demographics. In addition, the household questionnaire included an extensive module on IYCF practices. Following the WHO and UNICEF (2009) guidelines, anthropometric measurements (height, weight and middle‐upper arm circumference) were taken for children less than 24 months of age.

In the August 2017 round, the survey team also interviewed 249 HEWs working in the surveyed kebeles. Interviews in 15 kebeles could not be conducted because no HEW was present at the time of the visit or because the kebele did not have a health post.

### 2.3 Variables

We first assessed the extent to which the children 0–23 months in the sample followed age‐appropriate feeding practices. To do so, we considered six feeding indicators (WHO & UNICEF, 2021): (1) exclusive breastfeeding, (2) breastmilk and plain water, (3) breastmilk and other nonmilk liquids, (4) breastmilk and other milk, (5) breastmilk and complementary foods and (6) not breastfeeding. For this analysis, we used the data collected in March 2017 that also included children less than 6 months of age (the same children were 6 months or older in August 2017).

We then assessed the correlation between delayed introduction of complementary foods and future linear growth faltering. To this end, we constructed a binary indicator variable obtaining value 1 if the child 6–8 months of age did not consume complementary foods in the previous day in the March 2017 survey and zero otherwise. The outcome variable in this analysis was child's length for age Z‐score (LAZ) measured 6 months later in August 2017. We used the 2006‐WHO growth standards (de Onis et al., 2007; WHO, 2006) to convert child lengths to *Z*‐scores. Berhane et al. (2020) provide more details about the anthropometric measurements taken in this survey.

For all other analyses, we used the data from the August 2017 round in which both caregivers and local HEWs were interviewed. The survey instrument administered to the caregivers included a short test on their knowledge about IYCF practices (see supporting information S2). An identical test was included in the HEW survey instrument, permitting us to compare responses between caregivers and HEWs located in the same kebeles. We focused our analysis on the responses to the question that was formulated as follows: ‘At what age should a baby first start to receive foods (such as porridge) in addition to breast milk?’. The WHO (2013) recommends that after 6 months of exclusive breastfeeding, babies are transitioned to complementary foods (solid, semisolid or soft foods) because at this age, breastmilk alone cannot provide all nutrients required to support healthy growth and development. Therefore, we considered ‘6 months’ as the correct response to this question.

We also constructed a series of child, caregiver and household level controls for the regression analyses. Supporting information S3 provides more information about these variables.

### 2.4 Statistical analyses

The statistical analyses proceeded in stages. In the first stage, we assessed the prevalence of age‐appropriate IYCF practices in these localities. Using the March‐2017 survey data, we analysed age‐specific feeding practices for children 0–23 months.

In the second stage, we used unadjusted and adjusted linear regressions to assess the degree to which delayed introduction of complementary foods was associated with future linear growth faltering. To do so, we restricted the sample to children who were 6–8 months of age in March 2017. We then regressed child's length for age Z‐score (LAZ) measured in August 2017 on the binary indicator capturing children for whom the introduction of complementary foods was delayed in March 2017. The adjusted regressions further controlled for a series of child and household level characteristics, all measured in March 2017. Supporting information S4 provides the means and standard deviations of the variables used in this regression.

In the third stage, we calculated the proportion of caregivers and HEWs who responded correctly to the question about appropriate age when the children should be introduced to complementary foods.

In the last stage, we employed unadjusted and adjusted logistic regressions. We first tested the association between caregiver knowledge and the likelihood that a child 6–8 months consumed complementary foods in the previous day. The adjusted regressions controlled for observable confounding factors, such as child characteristics (age and sex), caregiver's characteristics (age and level of education) and household characteristics (head's characteristics, household demographics, wealth levels and distances to the nearest health post, water point and food market). Supporting information S5 provides the means and standard deviations of these variables. We then used the same regression approach with the same control variables to test the association between HEW's knowledge and the likelihood that a caregiver responded correctly to the question about the age when complementary foods should be introduced. Supporting information S6 provides the means and standard deviations of the variables used in this regression. The coefficients in these logistic regressions were reported as odds ratios.

In all regression analyses, the standard errors were clustered at the woreda level to account for the sampling design (Abadie, Athey, Imbens, & Wooldridge, 2017). All statistical analyses were conducted using Stata, version 16.1.

## 3 RESULTS

**Figure 1:**
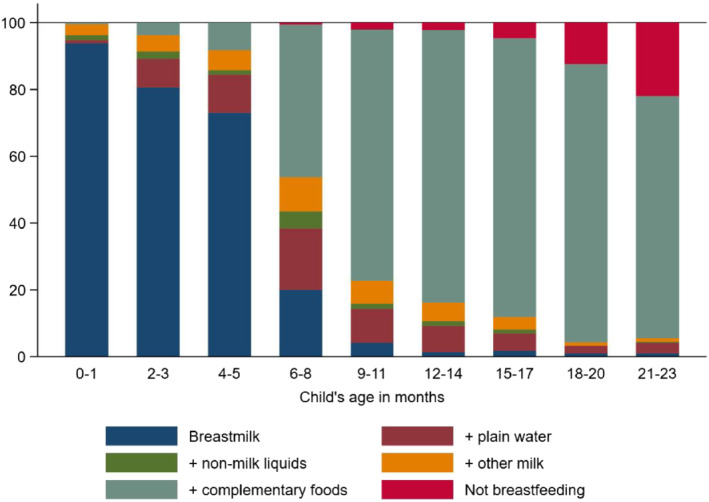
Infant and child feeding practices, by age. Note: *N* = 2596 children (0–23 months of age). Source: Authors' calculation from March‐2017 survey

Data from the March 2017 survey round indicated that the prevalence of age‐appropriate breastfeeding practices was relatively high (Figure 1). More than 90% of children 0–1 months were exclusively breastfed with the share falling to 81% and 73% for children in the 2‐ to 3‐month and 4‐ to 5‐month age groups, respectively. About 53% of the children 6–8 months of age did not consume complementary foods in the previous day. The share of children not consuming complementary foods declined by child's age but even at 12–14 months, 16% of the children did not consume solid, semisolid or soft foods in the previous day, only breastmilk and/or liquids.

Unadjusted and adjusted linear regressions showed that delayed introduction of complementary foods was negatively associated with lower LAZ score 6 months later (Table 1). Children 6–8 months who did not consume solid, semisolid or soft foods in the previous day in March 2017 had a 0.48 SD lower LAZ in August 2017 (*p* < 0.05), after controlling for a host of child, caregiver and household characteristics (column 2).

**Table 1:** Unadjusted and adjusted associations between delay in the introduction of complementary feeding and future growth faltering (LAZ in August 2017), linear regression

	(1)	2)
	*N* = 347	*N* = 345
Delayed complementary feeding	−0.329^*^	−0.482^**^
	(0.142)	(0.150)
*Child's characteristics:*		
Child's age in months		−0.218^*^
		(0.085)
Male child		−0.204
		(0.179)
Child was ill		−0.110
		(0.133)
*Mother's characteristics:*		
Mother's age (in years)		0.013
		(0.016)
Mother has no formal education		−0.416
		(0.254)
*Household characteristics:*		
Male headed household		0.577^**^
		(0.187)
Head age in years		−0.002
		(0.007)
Head has no formal education		0.160
		(0.244)
Household size		0.000
		(0.046)
Durable asset index		0.090^*^
		(0.042)
Number of tropical livestock units (TLU) owned		−0.011
		(0.017)
(ln) distance to the health post		−0.088
		(0.057)
(ln) distance to the nearest water point		0.130^*^
		(0.055)
(ln) distance from household to the nearest food market		0.163^**^
		(0.054)
Head is Orthodox		0.109
		(0.235)
Head is Muslim		−0.118
		(0.179)
Head follows other religion		(reference)
Binary control variables for each region?	No	Yes

*Note*: Linear regression. Outcome variable is length for age Z‐score (LAZ) measured in August 2017. All independent variables are measured using data from the March‐2017 survey round. ‘Delayed complementary feeding’ obtains value 1 if the child did not consume solid, semisolid or soft foods in the previous day (and zero if s/he did consume) in March 2017. The adjusted model (column 2) also includes binary control variables for each region (See supporting information S3 for more details). Sample restricted to children 6–8 months of age in March 2017. Standard errors reported in parentheses and clustered at the woreda level. Source: Authors' calculation from March and August 2017 surveys.

^+^ Statistical significance *p* < 0.10.

^*^ Statistical significance *p* < 0.05.

^**^ Statistical significance *p* < 0.01.

^***^ Statistical significance *p* < 0.001.

**Figure 2:**
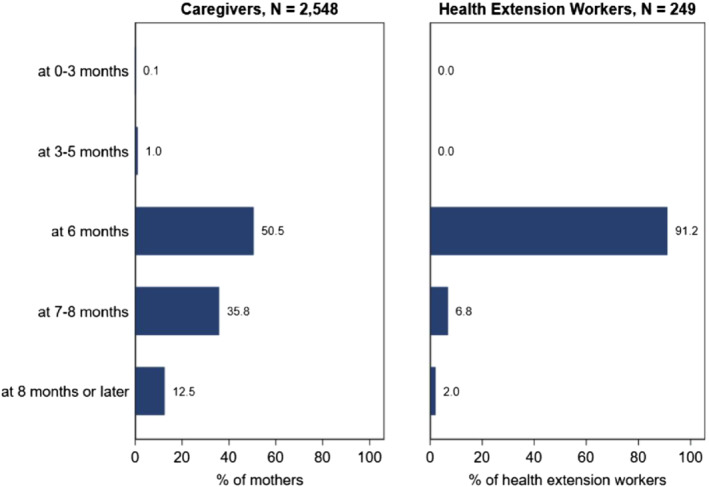
Caregiver and health extension worker responses to question ‘At what age should a baby first start to receive foods (such as porridge) in addition to breast milk?’. Note: *N* = 2548 caregivers and 249 Health Extension workers. Source: Authors' calculation from August‐2017 survey

Caregiver knowledge of age‐appropriate breastfeeding practices was high, but serious gaps were observed in age‐appropriate complementary feeding practices (supporting information S7). Half of the caregivers responded that complementary feeding should begin when children turn 6 months. However, a near‐equal proportion of the caregivers responded that the introduction should occur later (Figure 2). More than 90% of the HEWs responded that complementary feeding should begin when children turn 6 months while the remaining 10% responded that the CF should begin after 6 months (Figure 2).

Caregivers' responses about the age when complementary feeding should be introduced were strongly correlated with child feeding practices both in unadjusted and adjusted logistic regressions (Table 2). Column 2 of Table 2 shows that the likelihood that a child 6–8 months did not consume solid, semisolid or soft foods in the previous day was nearly 50% lower if the caregiver provided the correct response compared to children whose caregiver responded incorrectly (*p* < 0.05). Household wealth levels or the distance to the nearest food market was not associated with the likelihood that the child 6–8 months received complementary foods in the previous day (*p* > 0.05).

**Table 2:** Unadjusted and adjusted associations between caregiver's knowledge of age‐appropriate initiation of complementary feeding and the likelihood that the child 6–8 months did not consume solid, semisolid or soft foods in the previous day, odds ratios (OR) from logistic regressions

	(1)	(2)
	*N* = 294	*N* = 292
Caregiver responded correctly	0.534^*^	0.548^*^
	(0.148)	(0.157)
*Child's characteristics:*		
Child's age in months		0.467^***^
		(0.086)
Male child		1.019
		(0.265)
Child was ill		1.846^*^
		(0.569)
*Mother's characteristics:*		
Mother's age (in years)		1.017
		(0.032)
Mother has no formal education		1.277
		(0.532)
*Household characteristics:*		
Male headed household		1.960
		(0.993)
Head age in years		0.992
		(0.017)
Head has no formal education		0.822
		(0.349)
Household size		0.973
		(0.089)
Durable asset index		0.865^+^
		(0.070)
Number of tropical livestock units (TLU) owned		1.017
		(0.041)
(ln) distance to the health post		1.217
		(0.179)
(ln) distance to the nearest water point		1.038
		(0.147)
(ln) distance from household to the nearest food market		1.029
		(0.124)
Head is orthodox		0.461
		(0.374)
Head is Muslim		0.694
		(0.379)
Head follows other religion		(reference)
Binary control variables for each region?	No	Yes

*Note*: Logistic regression. Estimates are odds ratios (OR). Outcome variable obtains the value 1 if the child did not consume solid, semisolid or soft foods in the previous day (and zero if s/he did consume). The adjusted regression also includes binary control variables for each region (See supporting information S3 for more details). Sample restricted to children 6–8 months of age in August 2017. Estimates are odds ratios. Standard errors reported in parentheses and clustered at the woreda level. Source: Authors' calculation from August‐2017 survey.

^+^ Statistical significance *p* < 0.10.

^*^ Statistical significance *p* < 0.05.

^**^ Statistical significance *p* < 0.01.

^***^ Statistical significance *p* < 0.001.

Finally, Table 3 presents the odds ratios from a multivariable logistic regression where the dependent variable obtains a value one if the caregiver responded that CF should begin when the child turns 6 months and zero otherwise. The likelihood that the caregiver responded correctly was strongly and positively associated with the local HEW's response, and this association remained statistically significant when we controlled for various child, maternal and household characteristics. Column 2 of Table 3 shows that the correct response from the HEW nearly doubled the likelihood that the caregiver responded correctly to the question about the timing the introduction of complementary foods (*p* < 0.01).

**Table 3:** Unadjusted and adjusted associations between local health extension worker's knowledge and caregiver's knowledge of age‐appropriate initiation of complementary feeding, odds ratios (OR) from logistic regressions

	(1)	(2)
	*N* = 2402	*N* = 2375
HEW responded correctly	1.965^**^	1.867^**^
	(0.503)	(0.361)
*Child's characteristics:*		
Child's age in months		1.001
		(0.007)
Male child		0.993
		(0.068)
Child was ill		0.977
		(0.103)
*Mother's characteristics:*		
Mother's age (in years)		1.006
		(0.009)
Mother has no formal education		0.723^**^
		(0.090)
*Household characteristics:*		
Male headed household		1.066
		(0.172)
Head age in years		0.994
		(0.005)
Head has no formal education		1.091
		(0.147)
Household size		0.961
		(0.025)
Durable asset index		1.020
		(0.027)
Number of tropical livestock units (TLU) owned		1.005
		(0.013)
(ln) distance to the health post		1.073
		(0.050)
(ln) distance to the nearest water point		1.071+
		(0.042)
(ln) distance from household to the nearest food market		0.957
		(0.046)
Head is Orthodox		0.734
		(0.208)
Head is Muslim		0.837
		(0.213)
Head follows other religion		(reference)
Binary control variables for each region?	No	Yes

*Note*: Logistic regression. Estimates are odds ratios (OR). Outcome variable obtains the value 1 if the caregiver responded correctly (and zero if incorrectly). The adjusted regression also includes binary control variables for each region (See supporting information S3 for more details). Sample restricted to kebeles in which the HEW was interviewed. Estimates are odds ratios. Standard errors reported in parentheses and clustered at the woreda level. Source: Authors' calculation from August‐2017 survey.

Statistical significance denoted by

^+^ Statistical significance *p* < 0.10.

^*^ Statistical significance *p* < 0.05.

^**^ Statistical significance *p* < 0.01.

^***^ Statistical significance *p* < 0.001.

## 4 DISCUSSION

Exclusive breastfeeding for the first 6 months, followed by introduction of complementary foods at 6 months in addition to breastmilk has been a mainstay recommendation of complementary feeding guidelines (Lutter, Grummer‐Strawn, & Rogers, 2021; PAHO/WHO, 2003). The rationale behind this recommendation comes from evidence that the nutrient needs of full‐term, normal birth weight infants, can be met by human milk alone for the first 6 months (Kramer et al., 2001). Although some micronutrients can be limited in breastmilk (e.g., iron and zinc), the benefits related to preventing gastrointestinal infections outweighed the risks; hence, exclusive breastfeeding up to 6 months, but starting complementary feeding no later than 6 months, was recommended (PAHO/WHO, 2003). This recommendation takes into account the developmental readiness of children to start foods but also the insufficiency of breastmilk to supply energy and nutrient needs past 6 months of age (Lutter, Grummer‐Strawn, & Rogers, 2021).

In this sample of more than 2500 caregivers and their children, delays in the introduction of complementary feeding were widespread with more than 50% of the children 6–8 months not consuming solid, semisolid or soft foods in the previous day. This delay was shown to be associated with a higher risk of growth faltering in the second year of life, a reflection of the serious shortfall in energy and nutrients to support the growth and development of infants that solely rely on breastfeeding past 6 months of age (Allen & Dror, 2018; Dhami, Ogbo, Osuagwu, Ugboma, & Agho, 2019). Such serious shortfall in energy and nutrients is also likely to compromise immunity as well as brain development, leading to short and long‐term consequences (Cusick & Georgieff, 2016; Dewey & Brown, 2003). For example, delayed introduction to complementary (lumpy) foods was linked to lower diversity and long‐term feeding problems, such as difficulties in getting the child to eat and child becoming choosy with food later in life (Coulthard, Harris, & Emmett, 2009).

Our analysis suggests that the widespread delays in the introduction of complementary feeding are mainly due to misperceptions and limited knowledge among caregivers. Only 50% of the caregivers in our sample responded that complementary foods should be introduced at 6 months of age. Meanwhile, health workers' knowledge of age‐appropriate complementary feeding was high (~90%). In line with previous evidence from Ethiopia and elsewhere (Abebe, Haki, & Baye, 2016; Mbuya, Menon, Habicht, Pelto, & Ruel, 2013), local health workers knowledge predicted caregiver's knowledge about appropriate timing of complementary foods. However, the key constraint in transferring the knowledge from health workers to caregivers relates to limited contact between the two, as documented by previous research based on the same data (Abate, Dereje, Hirvonen, & Minten, 2020; Berhane et al., 2020). These knowledge gaps will need to be addressed by ensuring more frequent and timely visits by HEWs but also emphasis on not only what to feed but also when and how to feed the child (Baye, Tariku, & Mouquet‐Rivier, 2018). Indeed, increasing reach, intensity and frequency of age‐appropriate behavioural change communication have been shown to improve child feeding in various contexts (Frongillo, 2020; Menon et al., 2020). However, given challenges related to health workers multitasking (Mangham‐Jefferies, Mathewos, Russell, & Bekele, 2014), as well as the limited access to media platforms, innovative ways to deliver age‐appropriate nutrition messages are needed (Baye & Hirvonen, 2020a; CSA & ICF, 2016). Using frequent PSNP interactions as an additional platform to increase the intensity and reach of nutrition education could be explored further (UNICEF, MOLSA,,, & IFPRI, 2020). While health workers remain the primary source of nutrition messaging, it may also be important to strengthen the messaging using different platforms and to transmit the same message in a different way than otherwise done in public health where messaging tend to focus on ‘it is good for your baby’.

The present study has a number of strengths and limitations that need to be considered when interpreting our findings. First, our sampling of children from poor households from PSNP districts has likely led to a homogenous sample that could have underestimated the associations related to household wealth. Second, our study design does not allow us to establish causality; hence, findings should be interpreted as associations. However, the evaluation of nutrition knowledge of both health workers and caregivers, the large sample size and distribution as well as the multiple time points of the surveys uniquely position this study to inform future behavioural change communications that aim to improve nutrition in poor households.

## 5 CONCLUSIONS

The widespread delay in the introduction of complementary feeding in rural Ethiopia is likely to be an important contributor to the linear growth faltering among young children. Our analysis suggests that these delays are primarily due to caregiver knowledge gaps related to age‐appropriate complementary feeding practices. Our findings therefore highlight an urgent need to strengthen the effectiveness of behavioural change communication messaging within the health extension platforms and to explore innovative ways of increasing reach, intensity, and frequency of nutrition messaging. The nutrition sensitive PSNP provides a promising additional messaging platform through its reach of millions of rural families. But so far, implementation challenges coupled with resource gaps have hindered progress in achieving widespread positive impacts on diets and other nutritional outcomes (Berhane et al., 2020; UNICEF, MOLSA, et al., 2020). Finding solutions to these constraints forms an important task for researchers and policymakers.

## CONFLICT OF INTEREST

The authors declare that they have no conflicts of interest.

## CONTRIBUTIONS

KH and AW prepared and analyzed the data. KH and KB designed the research study with inputs from AL, SC and VV. KH and KB wrote the article with inputs from AL, SC and VV. All authors read and approved the final manuscript.

## Supporting information


**Data S1.** **Supplemental File S1:** Sampling
**Supplemental File S2:** Questionnaire module on knowledge about age appropriate IYCF practices
**Supplemental File S3:** Variable constructions
**Supplemental File S4:** Summary statistics of the variables in regression reported in Table 1
**Supplemental File S5:** Summary statistics of the variables in regression reported in Table 2
**Supplemental File S6:** Summary statistics of the variables in regression reported in Table 3
**Supplemental File S7:** Caregivers' knowledge on age appropriate infant and young child feeding
**Table S7.1** Perceptions on when to start breastfeeding (percent of mothers), N = 2,635
**Table S7.2** Perceptions on when to start complementary foods (percent of mothers), N = 2,548

## Data Availability

The data that support the findings of this study are available from the corresponding author upon reasonable request.
