# Supplementary material for: Understanding delays in the introduction of complementary foods in rural Ethiopia
Source: Matern Child Nutr. 2021 Sep 15;20(Suppl 5):e13247. doi: 10.1111/mcn.13247 (PMC11258766; doi:10.1111/mcn.13247)
Supplement: Supplementary file 1 — Data S1. Supplemental File S1: Sampling Supplemental File S2: Questionnaire module on knowledge about age appropriate IYCF practices Supplemental File S3: Variable constructions Supplemental File S4: Summary statistics of the variables in regression reported in Table 1 Supplemental File S5: Summary statistics of the variables in regression reported in Table 2 Supplemental File S6: Summary statistics of the variables in regression reported in Table 3 Supplemental File S7: Caregivers' knowledge on age appropriate infant and young child feeding Table S7.1 Perceptions on when to start breastfeeding (percent of mothers), N = 2,635 Table S7.2 Perceptions on when to start complementary foods (percent of mothers), N = 2,548 [file MCN-20-e13247-s001.docx]

**Understanding delays in the introduction of complementary foods in rural Ethiopia**

**Supplementary material**

## Supplemental File S1: Sampling

The sampling for this survey was done in stages. First, 88 woredas were randomly selected from the full list of woredas in which the Productive Safety Net Program (PSNP) operates in Amhara, Oromia, SNNP and Tigray regions. After this woreda selection, three kebeles were randomly selected from each woreda. The Central Statistical Agency (CSA) of Ethiopia divides each kebele into enumeration areas (EA) that are roughly equal in size, usually containing around 200 households. From each kebele, using the list of EAs provided to us by the CSA, one EA was randomly selected. Full household listing was carried out in all selected EAs in March 2017. Total of 46,866 households were listed residing in the EAs selected for the survey. Out of these, 10,318 households reported that they had a child less than 24 month of old.

Once the EA level census was completed, 10 eligible households from each EA were selected to be part of the survey. A household was eligible if it had a child less than 24-month-old. The sample was further stratified so that approximately half of the selected households were PSNP beneficiaries and half were poor (based on their own subjective assessment) but were not benefitting from the PSNP. Given the focus on poor PSNP and non-PSNP households, the sample is not representative of the EAs, kebeles or woredas from which the sample was drawn. In March, 2,635 households with a child less than 24 months were interviewed in 88 woredas, 264 kebeles in Amhara, Oromia, SNNP and Tigray regions. Out of these, 2,569 households were successfully revisited in August 2017, yielding an attrition rate of 2.5 percent.

The original purpose of the survey was to serve as a baseline to the evaluation of the nutrition sensitive components of the PSNP. Reflecting this, the sample size and the number of clusters were based on statistical power calculations and assumptions about attrition that permit the evaluation team to detect a 10 percentage point increase in child’s consumption of dairy products, a 12 percentage point increase in the likelihood that a mother has at least four antenatal care visits and a 10 percent increase in women’s body-mass index.

Finally, the survey teams also collected latitude-longitude coordinates of the household as well as the nearest food market and health post. In August round, the survey team also visited the health post and interviewed one health extension worker (HEW) working in the kebele. Most health posts in rural Ethiopia have two HEWs. Out of these, the enumerators interviewed the HEW that was available and willing to participate. A HEW interview was successfully conducted in 221 out of the 264 kebeles. In 43 kebeles, the HEW was not present either because the kebele did not have a health post or because none of the HEWs were present at time of interview. Berhane et al. (2020) provide further details about the survey.

## Supplemental File S2: Questionnaire module on knowledge about age appropriate IYCF practices

**Knowledge module administered to caregivers and health extension workers (see the next page for additional response options)**

| Q1. How long after birth should a baby start breastfeeding? **Code (a)** |
| --- |
| Q2. What should a mother do with the “first milk” or colostrum? **Code (b)** |
| Q3. Until what age should a baby by exclusively breastfed (only breastmilk, not even water?) |
|  |
| Q4. Why do you think that a baby under 6 months be exclusively breastfed? **Code (c)** Allow multiple responses |
| Q4b Until what age should a baby continue to be breastfed? |
|  |
| Q4c How often should a baby breastfeed?   1. Whenever baby wants; 2. When you see the baby is hungry, 3. When the baby cries, 4. Other 77. Don’t know |
| Q4d When the weather is very hot, should water be given to babies less than 6 months old?  1. Yes 2. No |
| Q5.At what age should a baby first start to receive liquids (including water) other than breast milk? |
|  |
| Q6. At what age should a baby first start to receive foods (such as porridge) in addition to breast milk? |
|  |
| Q6b At what age should a child be given the following foods? |
| 1. Water |
| 1. Injera, firfir, bread, etc. |
| 1. Legumes (shiro, pulses, peas) |
| 1. Green leafy vegetables (kale) |
| 1. Vegetables such as pumpkin, yam, carrots |
| 6.      Fruits such as bananas, papaya, mango |
| 1. Meat such as chicken, beef, goat, or fish |
| 1. Eggs |
| 1. Milk (cow, goat, camel, or powdered) |
| Q7. What can happen to children if they do not get enough iron (either in their diet or via iron supplements)?  **Code (d)** *Allow multiple responses*  *Enumerator: Please do not read the list of options. Ask the question as it is and recoded the response that you have got selecting from the lists.* |
| Q8. What are some foods that are rich in iron? **Code (e)** *Allow multiple responses*  *Enumerator: Please do not read the list of options. Ask the question as it is and recoded the response that you have got selecting from the lists.* |
| Q9. What can happen to children if they do not eat enough vitamin A-rich foods?  **Code (f)** *Allow multiple responses*  *Enumerator: Please do not read the list of options. Ask the question as it is and recoded the response that you have got selecting from the lists.* |
| Q10. What are some foods that contain vitamin A?  **Code (g)** *Allow multiple responses*  *Enumerator: Please do not read the list of options. Ask the question as it is and recoded the response that you have got selecting from the lists*. |

Additional response options:

| **Code (a)** | **Code (b)** | **Code (c)** | **Code (d)** |
| --- | --- | --- | --- |
| 1 Immediately, within 1 hour of delivery | 1 Give to baby by breastfeeding soon after birth | 1 Protects baby from illness/disease | 1 Impaired learning |
| 2 Some hours later but within 24 hours | 2 Throw away; start breastfeeding when real milk comes | 2 Breast milk contains everything a baby needs for the first 6 months | 2 Impaired development |
| 3 After 1 day | 3 Other | 3 Helps baby grow better | 3 Slow growth/lower height |
| 4 After 2 days | 77 Do not know | 4 Mother less likely to get pregnant | 4 Low immunity |
| 5 After more than 2 days |  | 5 Delays return of mother’s monthly bleeding | 5 Feel tired |
| 6 Do not think baby should be breastfed |  | 6 Breastmilk is clean, safe and convenient | 6 Become anemic |
| 77 Do not know |  | 7 Breastmilk is free/affordable | 7 Other |
|  |  | 8 Reduces health care cost | 88 Do not know |
|  |  | 9 Other |  |
|  |  | 88 Do not know |  |
|  |  |  |  |
| **Code (e)** | **Code (f)** | **Code (g)** |  |
| 1 Meat (beef, goat, etc.), chicken, fish | 1 Eye disease/vision problems | 1 Orange coloured fruits/vegetables |  |
| 2 Green leafy vegetables | 2 Low resistance to illness/diseases | 2 Green leafy vegetables |  |
| 3 Beans, peas | 3 Other | 3 Eggs |  |
| 4 Commercially fortified foods | 77 Do not know/ Remember | 4 Liver |  |
| 5 Other |  | 5 Breastmilk |  |
| 77 Do not know/ Remember |  | 6 Cow’s milk |  |
|  |  | 7 Other |  |
|  |  | 77 Do not know/ Remember |  |

## Supplemental File S3: Variable constructions

| **Variable** | **Note on construction** |
| --- | --- |
| **Dependent and key independent variables:** |  |
| Child did not consume solid, semi-solid and soft foods | Obtains a value 1 if child did not consume any solid, semi-solid and soft foods in the 24 hours prior to the interview, zero otherwise. |
| Mother responded correctly | Obtains a value 1 if mother responded correctly to the question about appropriate time when the children are introduced to complementary foods, zero otherwise. |
| HEW responded correctly | Obtains a value 1 if HEW at the kebele health post responded correctly to the question about appropriate time when the children are introduced to complementary foods, zero otherwise. |
| **Child characteristics:** |  |
| Child age | Child's age in months. |
| Male child | Obtains value 1 if male child, zero otherwise. |
| Child was ill | Obtains value 1 if child had fever or diarrhea in the 2 week period prior to the interview, zero otherwise. |
| **Caregiver characteristics (measured in March 2017):** |  |
| Mother's age | Mother's age in years. |
| Mother has no formal education | Obtains value 1 if mother has not gone to school, zero otherwise. |
| **Household characteristics (measured in March 2017):** |  |
| Male headed household | Obtains value 1 if male head, zero otherwise. |
| Head’s age | Household head’s age in years. |
| Head has no formal education | Obtains value 1 if head has not gone to school, zero otherwise. |
| Household size | Number of household members. |
| Durable asset index | Constructed using principal components methods |
| Tropical livestock units (TLUs) owned | Households' livestock holdings were converted to TLUs using conversion factors estimated in Jahnke (1982). |
| (ln) distance from household to the health post | Natural log of the distance (in kilometers) between the household’s location and the location of the health post. Calculated using latitude-longitude coordinates of the household and the health post. |
| (ln) distance from household to the nearest water point | Natural log of the time (in minutes) to fetch water and return (average over dry and rainy season) |
| (ln) distance from household to the nearest food market | Natural log of the distance (in kilometers) between the household’s location and the location of the nearest food market. Calculated using latitude-longitude coordinates of the household and the food market. |
| Head is Orthodox | Obtains value 1 if the household head is Orthodox, zero otherwise. |
| Head is Muslim | Obtains value 1 if the household head is Muslim, zero otherwise. |
| Head follows other religion (reference) | Obtains value 1 if the household head is not an Orthodox or a Muslim, zero otherwise. Used as the reference category in the regressions. |
| Located in the Amhara region | Obtains value 1 if the household is in the Amhara region, zero otherwise. |
| Located in the Oromia region | Obtains value 1 if the household is in the Oromia region, zero otherwise. |
| Located in the SNNP region | Obtains value 1 if the household is in the SNNP region, zero otherwise. |
| Located in the Tigray region (reference) | Obtains value 1 if the household is in the Tigray region, zero otherwise. Used as the reference category in the regressions. |

## Supplemental File S4: Summary statistics of the variables in regression reported in Table 1

|  | **N** | **mean** | **std. dev.** |
| --- | --- | --- | --- |
| Length-for-age Z-score measured in August 2017 (*dependent variable*) | 347 | -1.49 | 1.41 |
| Child did not consume solid, semi-solid and soft foods in March 2017 | 349 | 0.52 | 0.50 |
| **Child characteristics (all measured in March 2017):** |  |  |  |
| Child age | 349 | 7.03 | 0.85 |
| Male child | 349 | 0.46 | 0.50 |
| Child was ill | 349 | 0.38 | 0.49 |
| **Caregiver characteristics (all measured in March 2017):** |  |  |  |
| Mother's age | 349 | 28.87 | 6.33 |
| Mother has no formal education | 349 | 0.83 | 0.38 |
| **Household characteristics (all measured in March 2017):** |  |  |  |
| Male headed household | 349 | 0.90 | 0.30 |
| Head’s age | 349 | 37.89 | 11.23 |
| Head has no formal education | 349 | 0.75 | 0.43 |
| Household size | 349 | 5.86 | 1.89 |
| Durable asset index | 349 | 0.31 | 1.86 |
| Tropical livestock units (TLUs) owned | 349 | 3.14 | 4.75 |
| (ln) distance from household to the health post | 348 | 0.38 | 1.12 |
| (ln) distance from household to the nearest water point | 348 | 3.40 | 1.27 |
| (ln) distance from household to the nearest food market | 349 | 1.49 | 1.40 |
| Head is Orthodox | 349 | 0.50 | 0.50 |
| Head is Muslim | 349 | 0.32 | 0.47 |
| Head follows other religion (*reference* *category*) | 349 | 0.18 | 0.39 |
| Located in the Amhara region | 349 | 0.21 | 0.41 |
| Located in the Oromia region | 349 | 0.28 | 0.45 |
| Located in the SNNP region | 349 | 0.24 | 0.43 |
| Located in the Tigray region (*reference* *category*) | 349 | 0.28 | 0.45 |

*Note: Sample restricted to children 6-8 months of age in March 2017. All data originate from March 2017 round.*

## Supplemental File S5: Summary statistics of the variables in regression reported in Table 2

|  | **N** | **mean** | **std. dev.** |
| --- | --- | --- | --- |
| Child did not consume solid, semi-solid and soft foods (*dependent variable*) | 295 | 0.55 | 0.50 |
| Mother responded correctly | 294 | 0.49 | 0.50 |
| **Child characteristics:** |  |  |  |
| Child age | 295 | 6.93 | 0.79 |
| Male child | 295 | 0.53 | 0.50 |
| **Caregiver characteristics:** |  |  |  |
| Mother's age | 295 | 28.23 | 6.26 |
| Mother has no formal education | 295 | 0.85 | 0.36 |
| **Household characteristics:** |  |  |  |
| Male headed household | 295 | 0.90 | 0.30 |
| Head’s age | 295 | 36.66 | 9.92 |
| Head has no formal education | 295 | 0.85 | 0.36 |
| Household size | 295 | 5.85 | 2.00 |
| Durable asset index | 294 | 0.13 | 1.76 |
| Tropical livestock units (TLUs) owned | 295 | 2.86 | 3.19 |
| (ln) distance from household to the health post | 295 | 0.47 | 1.00 |
| (ln) distance from household to the nearest water point | 294 | 3.33 | 1.18 |
| (ln) distance from household to the nearest food market | 295 | 1.47 | 1.28 |
| Head is Orthodox | 295 | 0.45 | 0.50 |
| Head is Muslim | 295 | 0.33 | 0.47 |
| Head follows other religion (*reference* *category*) | 295 | 0.22 | 0.42 |
| Located in the Amhara region | 295 | 0.25 | 0.43 |
| Located in the Oromia region | 295 | 0.28 | 0.45 |
| Located in the SNNP region | 295 | 0.25 | 0.44 |
| Located in the Tigray region (*reference* *category*) | 295 | 0.21 | 0.41 |

*Note: Sample restricted to children 6-8 months of age in August 2017. All data originate from August 2017 round.*

## Supplemental File S6: Summary statistics of the variables in regression reported in Table 3

|  | **N** | **mean** | **std. dev.** |
| --- | --- | --- | --- |
| Mother responded correctly (*dependent variable*) | 2,402 | 0.51 | 0.50 |
| Local health extension worker (HEW) responded correctly | 2,422 | 0.91 | 0.28 |
| **Child characteristics:** |  |  |  |
| Child age | 2,422 | 16.94 | 6.90 |
| Male child | 2,422 | 0.50 | 0.50 |
| **Caregiver characteristics:** |  |  |  |
| Mother's age | 2,420 | 28.89 | 6.49 |
| Mother has no formal education | 2,422 | 0.84 | 0.37 |
| **Household characteristics:** |  |  |  |
| Male headed household | 2,422 | 0.89 | 0.31 |
| Head’s age | 2,422 | 38.02 | 10.77 |
| Head has no formal education | 2,422 | 0.81 | 0.40 |
| Household size | 2,422 | 5.78 | 1.96 |
| Durable asset index | 2,421 | 0.27 | 1.80 |
| Tropical livestock units (TLUs) owned | 2,422 | 3.11 | 3.58 |
| (ln) distance from household to the health post | 2,412 | 0.46 | 1.05 |
| (ln) distance from household to the nearest water point | 2,408 | 3.39 | 1.19 |
| (ln) distance from household to the nearest food market | 2,422 | 1.52 | 1.30 |
| Head is Orthodox | 2,422 | 0.49 | 0.50 |
| Head is Muslim | 2,422 | 0.31 | 0.46 |
| Head follows other religion (*reference* *category*) | 2,422 | 0.20 | 0.40 |
| Located in the Amhara region | 2,422 | 0.24 | 0.43 |
| Located in the Oromia region | 2,422 | 0.24 | 0.43 |
| Located in the SNNP region | 2,422 | 0.26 | 0.44 |
| Located in the Tigray region (*reference* *category*) | 2,422 | 0.26 | 0.44 |

*Note:* *Sample restricted to kebeles in which the HEW was interviewed in August 2017. All data originate from August 2017 round.*

## Supplemental File S7: Caregivers’ knowledge on age appropriate infant and young child feeding

Caregivers were tested about their knowledge of age appropriate IYCF practices. Table S9.1. reports on their knowledge regarding age-appropriate breastfeeding practices using data collected in the March 2017 survey. The World Health Organization recommends that breastfeeding is initiated within the first hour after the child is born (WHO, 2013). Nearly 80 percent of the caregivers in our sample agreed with this (Table S7.1.) by responding that breastfeeding should begin immediately after the baby is born.

The World Health Organization further recommends that babies are introduced to complementary food at 6 months of age (WHO, 2013). The data collected in August-2017 shows that only about 50 % of the caregivers agreed with this guideline (Table S7.2). About 36 % responded that the appropriate age is 7-8 months and nearly 13 percent responded that the ideal age is at 8 months or later.

Table S7.1 Perceptions on when to start breastfeeding (percent of mothers), N=2,635

| **Caregiver response** | **%** |
| --- | --- |
| Immediately, within 1 hour | 79.6 |
| Some hours later but within 24 hours | 14.5 |
| After 1 day | 2.2 |
| After 2 days | 0.6 |
| After more than 2 days | 1.1 |
| Baby should not be breastfed | 0.5 |
| Do not know | 1.5 |
| Total | **100** |

*Source: Authors’ calculation from March-2017 survey.*

Table S7.2 Perceptions on when to start complementary foods (percent of mothers), N=2,548

| **Caregiver response** | **%** |
| --- | --- |
| at 0-3 months | 0.1 |
| at 3-5 months | 1.0 |
| at 6 months | 50.5 |
| at 7-8 months | 35.8 |
| at 8 months or later | 12.5 |
| Total | **100** |

*Source: Authors’ calculation from August-2017 survey.*

## Supplement references

Berhane, G., Golan, J., Hirvonen, K., Hoddinott, J., Kim, S., Taffesse, A. S., . . . Yimer, F. (2020). *Evaluation of the Nutrition-sensitive Features of the Fourth Phase of Ethiopia's Productive Safety Net Programme*. Addis Ababa: International Food Policy Research Institute (IFPRI), Ethiopia Strategy Support Program (ESSP).

Jahnke, H. E. (1982). *Livestock production systems and livestock development in tropical Africa* (Vol. 35): Kieler Wissenschaftsverlag Vauk Kiel.

WHO. (2013). *Essential nutrition actions: improving maternal, newborn, infant and young child health and nutrition* (9241505559). Geneva: World Health Organization (WHO).
